# Uric acid and HDL-C-derived inflammatory ratios as biomarkers in schizophrenia and major depressive disorder: a retrospective study

**DOI:** 10.3389/fpsyt.2025.1698455

**Published:** 2025-11-03

**Authors:** Hao Chen, Min Qiu, Haiqing Zhang, Yan Huai, Qiuyan Huang, Ping Li, Yingjia Lei, Yufeng Jiang, Qifeng Li, Shaochuan Zhang

**Affiliations:** Department of Clinical Laboratory, The Mental Hospital of Yunnan Province/The Affiliated Mental Health Center of Kunming Medical University, Yunnan, Kunming, China

**Keywords:** schizophrenia, major depressive disorder, inflammatory biomarkers, oxidative stress, uric acid, high-density lipoprotein cholesterol

## Abstract

**Background:**

Ratios derived from uric acid (UA) and high-density lipoprotein cholesterol (HDL-C) represent novel composite indicators integrating metabolic and inflammatory information. These ratios include the uric acid-to-lymphocyte ratio (ULR), uric acid-to-HDL-C ratio (UHR), uric acid-to-creatinine ratio (UCR), uric acid-to-albumin ratio (UAR), neutrophil-to-HDL-C ratio (NHR), lymphocyte-to-HDL-C ratio (LHR), monocyte-to-HDL-C ratio (MHR), and platelet-to-HDL-C ratio (PHR). This retrospective study aimed to evaluate and compare the predictive ability of these indicators in distinguishing patients with schizophrenia (SCZ) or major depressive disorder (MDD) from healthy controls (HCs) to identify potential biomarkers.

**Materials and methods:**

Blood parameter data were collected from 442 patients with SCZ, 326 with MDD, and 222 healthy controls. A retrospective analysis was conducted to examine intergroup differences in the UA- and HDL-C-derived ratios. The predictive efficacy of these parameters was assessed using receiver operating characteristic (ROC) curve analysis.

**Results:**

Significant intergroup differences in biomarker levels were observed. The UHR, UAR, NHR, and MHR were identified as predictive factors for distinguishing the SCZ group from the HC group, while the UAR and MHR distinguished the MDD group from the HC group. A composite model of the UHR, UAR, NHR, and MHR in the SCZ group yielded an area under the curve (AUC) of 0.877 (p<0.001). Similarly, a composite model of the UAR, UCR, UHR, and MHR in the MDD group produced an AUC of 0.818 (p<0.001).

**Conclusion:**

This study indicated that the ratio derived from UA and HDL-C more comprehensively reflects the inflammatory and metabolic status, and its expression differences can significantly distinguish SCZ and MDD.

## 1 Introduction

Schizophrenia (SCZ) and major depressive disorder (MDD) are two major causes of disability worldwide. They are characterized by psychotic symptoms and a persistently low mood, respectively. Although drug treatment can effectively control symptoms, it is only moderately effective and poorly tolerated. Patients are prone to relapse shortly after medication is discontinued (1, 2). Therefore, breakthrough treatment options are urgently needed. Recent survey research found that the weighted lifetime prevalence of SCZ in China was 0.6%, while the corresponding figure for MDD in adults was 3.4% (3). Patients with SCZ had an unusually short life expectancy, with high mortality rates across all age groups, resulting in an expected lifespan that was approximately 20 years shorter than that of the general population (4). Meta-analytic studies indicated that the risk of suicide in patients with MDD was 8.62 times higher than that of the general population (5). However, the etiology of these two disorders remains unclear, and research into novel biomarkers is ongoing.

In recent years, research on inflammatory markers in the diagnosis and treatment of mental disorders has provided a new perspective. Studies have found that patients with depression exhibit systemic immune activation, and their levels of inflammatory markers, immune cell counts, and antibody titers are all in an abnormal state (6, 7). Novel classic hematological cell parameters (such as white blood cell count, neutrophil percentage, monocyte percentage, NLR, PLR, etc.) serve as inflammatory markers and hold significant value in the disease prognosis (8), treatment response (9), and suicide risk prediction (10) of patients with schizophrenia spectrum disorders and depression. They can also effectively distinguish between patients with treatment-resistant schizophrenia (TRS) and non-treatment-resistant schizophrenia (NTRS) (11). Furthermore, the interplay between inflammation and oxidative stress plays a crucial role in the pathophysiology of specific neuropsychiatric disorders such as MDD, anxiety, SCZ, and autism (12). For instance, unconjugated bilirubin (UCB) was a potent endogenous plasma antioxidant, and the model constructed by combining UCB with inflammatory markers (MLR, MPV, PLR) can serve as a simple and effective indicator for identifying acute schizophrenia (13). In recent years, the key role of uric acid (UA) and high-density lipoprotein cholesterol (HDL-C) in regulating inflammatory responses and alleviating oxidative stress (14, 15) has revealed the possibility of combining them with classic hematological cell parameters as potential markers for inflammation and oxidative stress metabolism.

The uric acid/lymphocyte ratio (ULR), uric acid/high-density lipoprotein cholesterol ratio (UHR), uric acid/creatinine ratio (UCR), uric acid/albumin ratio (UAR), neutrophil/HDL-C ratio (NHR), lymphocyte/HDL-C ratio (LHR), monocyte/HDL-C ratio (MHR), and platelet/HDL-C ratio (PHR) have been identified as potential biomarkers of systemic inflammation and oxidative stress in various inflammatory diseases (16–20). However, there is currently limited research on these ratios in the context of mental disorders. Some studies have revealed the role of individual indicators related to UA or HDL-C ratios (16, 20, 21). To our knowledge, there have been no large-scale studies assessing the differences in ULR, UHR, UCR, UAR, NHR, LHR, MHR, and PHR values between patients with SCZ and those with MDD, nor has the association mechanism of these inflammatory factors with SCZ and MDD been clearly defined. We hypothesize that these inflammatory markers are correlated with SCZ and MDD to varying degrees. Therefore, this study aims to utilize large-scale clinical data to evaluate the following questions: 1) Are there differences in the aforementioned indicators between patients with acute SCZ, MDD, and healthy individuals? 2) Can these indicators serve as potential biomarkers?

## 2 Materials and methods

### 2.1 Study design and population

This retrospective analysis included patients diagnosed with SCZ or MDD who were admitted to Yunnan Provincial Mental Health Hospital for treatment due to acute episodes between January 2024 and January 2025. The control group comprised healthy individuals who underwent physical examinations at the same hospital during the same period and who had no history of psychiatric diagnosis or treatment. Sociodemographic and hematological data were extracted from the electronic medical record system for evaluation. This study utilized only the initial admission records from the inpatient ward at the time of admission for each patient. Blood test information was obtained from the first set of blood tests conducted upon admission, which was typically performed in the early morning on the second day after admission in patients who had fasted. It should be noted that all cases involved patients who had not received effective treatment outside the hospital due to poor medication response or adherence within one month, and required hospitalization for acute exacerbations. The inclusion criteria for participants were as follows: (a) met the diagnostic criteria according to the ICD-10 at the time of admission; (b) had complete blood count and biochemical test results; and (c) were between 19 and 65 years of age. The exclusion criteria were as follows: (a) individuals with any of the following conditions: severe infection, hypertension, diabetes, autoimmune diseases, heart failure, traumatic brain injury, epilepsy, malignant tumors, or other chronic diseases requiring medication. (b) individuals with significantly elevated or reduced white blood cell (WBC) and platelet counts, as indicated by leukocytosis (>10×10^9^ cells/L), leukopenia (<4×10^9^ cells/L), thrombocytosis (>450×10^9^ cells/L), or thrombocytopenia (<100×10^9^ cells/L), to reduce the risk of enrolling patients with severe inflammatory diseases. The screening criteria for healthy participants were as follows: (a) aged 19 to 65 years; (b) having complete biochemical and routine blood test results; and (c) having no history of medication use. The exclusion criteria for the healthy control group were as follows: (a) individuals with mental disorders or severe acute or chronic diseases (e.g., infections, autoimmune diseases, heart failure, head injuries, epilepsy, or tumors); and (b) individuals with leukocytosis, leukopenia, thrombocytosis, or thrombocytopenia.

### 2.2 Data collection and calculation

Clinical data was systematically retrieved from electronic medical records and laboratory information systems. Venous blood samples were collected during the standardized time window of 06:00 to 07:30, adhering to established clinical protocols. Subsequent laboratory analyses were conducted by certified technicians to generate comprehensive hematological and biochemical profiles. The following parameters were extracted for analysis: UA level, creatinine level, albumin level, alanine aminotransferase (ALT), and glutamate aminotransferase (AST) levels, leukocyte count, neutrophil count, lymphocyte count, monocyte count, platelet count, total cholesterol (TC) level, triglycerides (TG) level, HDL-C level, and low-density lipoprotein cholesterol (LDL-C) level. Derived biomarkers were calculated using specific formulas: the UHR was determined by dividing the UA concentration by the lymphocyte count; the UAR was computed by dividing the UA level by the albumin level; and the UCR was established by dividing the UA concentration by the creatinine concentration. Additionally, HDL-C-based ratios were calculated as follows: the UHR by dividing the UA level by the HDL-C level; the NHR by dividing the neutrophil count by the HDL-C level; the LHR by dividing the lymphocyte count by the HDL-C level; the MHR by dividing the monocyte count by the HDL-C level; and the PHR by dividing the platelet count by the HDL-C level.

### 2.3 Ethical considerations

This low-risk retrospective observational study was approved by the Ethics Committee of Kunming Medical University Mental Health Center (approval number: YNJS-20230615-001). Owing to the low risk of this study, written informed consent was waived, and all the data were anonymized to ensure confidentiality.

### 2.4 Statistical analysis

Statistical analyses were conducted using SPSS version 30.0. Continuous variables were presented as the mean ± standard deviation, while categorical variables were expressed as frequencies and percentages. The normality of the data distribution was assessed using the Kolmogorov-Smirnov test. For non-normally distributed data with unequal variances, the Kruskal-Wallis test was applied, whereas one-way analysis of variance (ANOVA) was used for normally distributed data. Categorical data was analyzed using the chi-square test (χ²). Intergroup differences were examined through *post hoc* analyses with Bonferroni correction. Relationships between variables were evaluated using Spearman’s correlation analysis. Univariate analysis was performed to identify potential disease risk factors, followed by multifactorial logistic regression incorporating variables with statistical significance (p<0.05). Confounding variables, including sex and age, were adjusted for using binary logistic regression. Receiver operating characteristic (ROC) curve analysis was employed to assess diagnostic factors; with optimal thresholds determined using Youden’s index. Diagnostic performance was evaluated through a combined measurement model constructed via binary logistic regression. Highly variable MHR values were minimized by multiplying them by 10 (22). Effect sizes are reported as odds ratios (ORs) with corresponding 95% confidence intervals (CIs). Statistical significance was defined as p<0.05.

## 3 Results

### 3.1 Demographic and clinical characteristics of participants

In this study, data from a total of 474 patients with SCZ, 361 patients with MDD, and 283 healthy individuals were extracted from the electronic medical records system. After participants with incomplete data were excluded, the analysis included 442 patients with SCZ, 326 patients with MDD, and 222 healthy individuals. As shown in **Table 1**, the mean age of the patients in the SCZ group was 35.735 ± 11.766 years; it included 177 males and 265 females. The mean age of the MDD group was 35.758 ± 13.254 years (102 males and 224 females), and the mean age of the HC group was 35.824 ± 6.704 years (42 males and 180 females). Statistical analysis revealed no statistically significant difference in age between the patient group and the HC group (p > 0.05) but revealed a statistically significant difference in sex (p <0.05). The percentages of males in the SCZ, MDD, and HC groups were 40.0%, 31.3%, and 18.9%, respectively, and the percentages of females were 60.0%, 68.7% and 81.1%, respectively. 3.2 Comparison of Laboratory Indicators and Biomarker Analysis Results Between Patients with Schizophrenia or Major Depressive Disorder and Healthy Controls.

**Table 1:** Comparison of demographic features and laboratory indicators between schizophrenia (SCZ), major depressive disorder (MDD), and healthy controls (HCs).

Variables	SCZ group (n=442)	MDD group (n=326)	HC group (n=222)	H/χ2	*P*
Age (year)	35.735 ± 11.766	35.758 ± 13.254	35.824 ± 6.704	4.563	0.102
Sex (male/female)	177/265	102/224	42/180	30.388	**<0.05**
WBC (10^9^/L)	6.954 ± 2.146**^a^**,**^b^**	5.995 ± 1.518	5.810 ± 1.274	65.191	**<0.001**
ALT (U/L)	23.751 ± 35.092**^a^**,**^b^**	17.731 ± 16.620	16.568 ± 9.480	27.704	**<0.001**
AST (U/L)	23.357 ± 15.567**^b^**	18.920 ± 8.908**^a^**	20.126 ± 5.140	45.265	**<0.001**
Urea (mmol/L)	4.369 ± 1.440**^a^**	4.427 ± 1.296**^a^**	4.750 ± 1.128	23.860	**<0.001**
Neutrophil (10^9^/L)	1.171 ± 0.254**^a^**,**^b^**	1.215 ± 0.253	1.330 ± 0.288	54.487	**<0.001**
Lymphocyte (10^9^/L)	2.311 ± 0.743**^a^**,**^b^**	2.133 ± 0.621	2.104 ± 0.579	16.192	**<0.001**
Monocyte (10^9^/L)	0.531 ± 0.189**^a^**,**^b^**	0.459 ± 0.148**^a^**	0.393 ± 0.114	105.759	**<0.001**
Platelet (10^9^/L)	257.706 ± 63.329	251.979 ± 60.656	257.315 ± 48.535	2.266	0.322
TC (mmol/L)	4.149 ± 0.878**^a^**	4.253 ± 0.822**^a^**	4.444 ± 0.748	25.335	**<0.001**
TG (mmol/L)	1.455 ± 0.844	1.543 ± 1.372	1.281 ± 0.658	5.341	0.069
HDL-C (mmol/L)	1.171 ± 0.254**^a^**,**^b^**	1.215 ± 0.253**^a^**	1.330 ± 0.288	51.420	**<0.001**
LDL-C (mmol/L)	3.049 ± 0.691**^a^**,**^b^**	2.511 ± 0.715	2.575 ± 0.639	120.044	**<0.001**
Uric acid (μmol/L)	392.131 ± 105.404**^a^**,**^b^**	346.515 ± 97.689	324.162 ± 49.235	84.403	**<0.001**
Creatinine (μmol/L)	62.894 ± 12.946	61.797 ± 13.294	61.615 ± 11.244	2.261	0.323
Albumin(g/L)	42.263 ± 9.223**^a^**,**^b^**	40.740 ± 3.570**^a^**	46.735 ± 2.761	296.319	**<0.001**
ULR	186.362 ± 82.986	175.307 ± 69.166	164.576 ± 47.422	5.944	0.051
UHR	352.582 ± 123.821**^a^**,**^b^**	302.323 ± 123.032**^a^**	257.332 ± 75.175	101.517	**<0.001**
UAR	9.360 ± 2.403**^a^**,**^b^**	8.514 ± 2.286**^a^**	6.951 ± 1.070	187.456	**<0.001**
UCR	6.333 ± 1.605**^a^**,**^b^**	5.705 ± 1.520	5.369 ± 0.976	71.408	**<0.001**
NHR (10^9^/mmol)	3.500 ± 1.775**^a^**,**^b^**	2.742 ± 1.194	2.484 ± 0.926	80.273	**<0.001**
LHR (10^9^/mmol)	2.076 ± 0.834**^a^**,**^b^**	1.843 ± 0.690**^a^**	1.659 ± 0.598	46.481	**<0.001**
MHR (10^9^/mmol)	0.477 ± 0.208**^a^**,**^b^**	0.397 ± 0.163**^a^**	0.311 ± 0.119	137.158	**<0.001**
PHR (10^9^/mmol)	229.830 ± 73.338**^a^**,**^b^**	215.492 ± 67.537	202.753 ± 60.071	24.090	**<0.001**

SCZ, schizophrenia; MDD, major depressive disorder; HC, healthy control; WBC, white blood cells; ALT, Alanine aminotransferase; AST, Glutamate aminotransferase; TC, Total cholesterol; TG, Triglycerides; HDL-C, High-density lipoprotein cholesterol; LDL-C, Low-density lipoprotein cholesterol; ULR, Uric acid/lymphocyte ratio; UHR, Uric acid/HDL-C ratio; UAR, Uric acid/Albumin ratio; UCR, Uric acid/Creatinine ratio; NHR, neutrophil/HDL-C ratio; LHR, Lymphocyte/HDL-C ratio; MHR, Monocyte/HDL-C ratio; PHR, Platelet/HDL-C ratio. a, vs. HC group, p<0.05; b, vs. MDD group, p<0.05. Values are represented as mean ± SEM. Bold values indicated statistical significance.

Statistical analysis showed that the levels of HDL-C, TC, and albumin in the case group were significantly lower than those in the HC group (p<0.05), suggesting the presence of lipid metabolism abnormalities in patients. The UA levels were significantly higher in the SCZ group compared to the MDD and HC group (p<0.05). Furthermore, the SCZ group exhibited more abnormal indicators compared to the MDD group, indicating that the schizophrenia patients in this study may have stronger inflammation or metabolic disturbances than those with MDD. Specifically, compared to the HC group, the SCZ group had higher counts of leukocytes, lymphocytes, and monocytes, as well as elevated levels of ALT, AST, LDL-C, UA, UHR, UAR, UCR, NHR, LHR, MHR, and PHR (p<0.05); it also had lower neutrophil counts; and lower levels of urea, TC, HDL-C, and albumin (p<0.05). In comparison with the HC group, the MDD group showed higher monocyte counts and elevated levels of UHR, UAR, LHR, and MHR (p<0.05), as well as lower levels of urea, AST, TC, HDL-C, and albumin (p<0.05).

**Figure 1:**
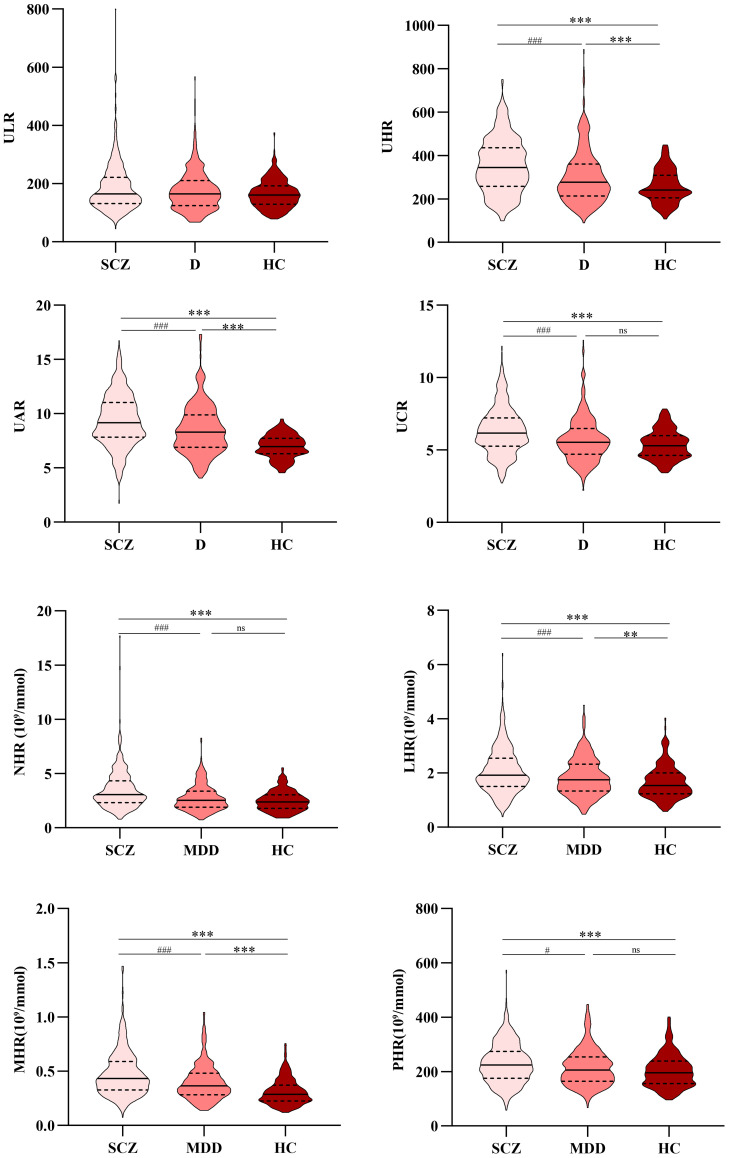
Comparison of ULR, UHR, UAR, UCR, NHR, LHR, MHR, and PHR among the SCZ, MDD, and HC groups. *, vs. HC group, p<0.05; #, vs. MDD group, p <0.05. SCZ, Schizophrenia; MDD, Major depressive disorder; HC, Healthy control; ULR, Uric acid/lymphocyte ratio; UHR, Uric acid/HDL-C ratio; UAR, Uric acid/Albumin ratio; UCR, Uric acid/Creatinine ratio; NHR, Neutrophil/HDL-C ratio; LHR, Lymphocyte/HDL-C ratio; MHR, Monocyte/HDL-C ratio; PHR, Platelet/HDL-C ratio. **, ***, vs. HC group, p<0.01; ##,###, vs. MDD group, p <0.01.

Compared to the MDD group, the SCZ group exhibited higher counts of leukocytes, lymphocytes, and monocytes, as well as elevated levels of ALT, AST, LDL-C, UA, albumin, UHR, UAR, UCR, NHR, LHR, MHR, and PHR; conversely, the neutrophil count and HDL-C level were lower (p <0.05). **Table 1** and **Figure 1** summarize the detailed results of all variables.

### 3.3 Correlation analysis of uric acid with other biomarkers

Spearman’s correlation analysis was employed to examine the relationships among laboratory parameters. Notably, across the three groups, UA exhibited positive correlations with white blood cells, ALT, lymphocytes, monocytes, triglycerides, creatinine, albumin, and various inflammatory ratios, while demonstrating a negative correlation with age and HDL-C. However, with the exception of creatinine, the correlation coefficients |r| were all less than 0.4. The correlation coefficients are presented in **Table 2**.

**Table 2:** Correlation coefficients between uric acid and other biomarkers.

Variables	Uric acid (μmol/L)		
	HC group	MDD group	SCZ group
Age (year)	-.158*	-.149**	-.254**
WBC (10^9^/L)	.183**	.158**	.234**
ALT (U/L)	.159*	.314**	.289**
AST (U/L)	0.019	.208**	.248**
Urea (mmol/L)	.138*	.153**	0.068
Neutrophil (10^9^/L)	0.084	.123*	.158**
Lymphocyte (10^9^/L)	.197**	.135*	.185**
Monocyte (10^9^/L)	.173**	.151**	.307**
Platelet (10^9^/L)	0.108	0.005	.168**
TC (mmol/L)	0.051	0.064	.154**
TG (mmol/L)	.257**	.308**	.200**
HDL-C (mmol/L)	-.279**	-.294**	-.205**
LDL-C (mmol/L)	.197**	.219**	-0.088
Creatinine (μmol/L)	.394**	.455**	.453**
Albumin(g/L)	.165*	.277**	.311**
NHR (10^9^/mmol)	.207**	.220**	.228**
LHR (10^9^/mmol)	.326**	.240**	.256**
MHR (10^9^/mmol)	.289**	.266**	.337**
PHR (10^9^/mmol)	.255**	.154**	.255**

Results given as Spearman correlation coefficient. SCZ, Schizophrenia; MDD, Major depressive disorder; HC, Healthy control; WBC, White blood cells; ALT, Alanine aminotransferase; AST, Glutamate aminotransferase; TC, Total cholesterol; TG, Triglycerides; HDL-C, High-density lipoprotein cholesterol; LDL-C, Low-density lipoprotein cholesterol; NHR, Neutrophil/HDL-C ratio; LHR, Lymphocyte/HDL-C ratio; MHR, Monocyte/HDL-C ratio; PHR, Platelet/HDL-C ratio. * p <0.05, ** p <0.01. All associations were statistically significant. Bold values indicated statistical significance.

### 3.4 Correlations among neutrophil, lymphocyte, monocyte, platelet counts, and lipids

Spearman’s correlation was used to analyze the correlations among the neutrophil count, lymphocyte count, monocyte count, platelet count, and lipid profiles in each group, as shown in **Table 3**. The neutrophil count in the HC group was positively correlated with LDL-C level and negatively correlated with the HDL-C level (p<0.05). The lymphocyte count was positively correlated with TG level (p<0.05). The monocyte count was positively correlated with LDL-C level (p<0.05). The platelet count was positively correlated with both TC and LDL-C levels (p<0.05). In the SCZ group, the neutrophil, lymphocyte, monocyte, and platelet counts were positively correlated with TC level, and the lymphocyte and monocyte counts were positively correlated with TG level and negatively correlated with HDL-C level (p<0.05). In the MDD group, the neutrophil count was positively correlated with TC, TG, and LDL-C levels. Lymphocyte and monocyte counts were negatively correlated with the HDL-C level (p <0.05). It was worth noting that the correlation coefficients |r| were all less than 0.3. The correlation coefficients are shown in **Table 3**.

**Table 3:** Correlations among neutrophil, lymphocyte, monocyte, platelet, and lipid profile indicators.

Parameters	Neutrophil (10^9^/L)	Lymphocyte (10^9^/L)	Monocyte (10^9^/L)	Platelet (10^9^/L)
HC group				
TC (mmol/L)	0.018	0.071	0.056	.166^*^
TG (mmol/L)	0.071	.165^*^	0.057	0.081
HDL-C (mmol/L)	-.152^*^	-0.025	-0.071	-0.056
LDL-C (mmol/L)	.173^**^	0.130	.150^*^	.214^**^
SCZ group				
TC (mmol/L)	.136^**^	.118^*^	.106^*^	.149^**^
TG (mmol/L)	0.078	.193^**^	.117^*^	0.058
HDL-C (mmol/L)	-0.047	-.127^**^	-.107^*^	-0.008
LDL-C (mmol/L)	0.043	-0.015	-0.018	-0.042
MDD group				
TC (mmol/L)	.134^*^	-0.096	-0.041	0.019
TG (mmol/L)	.111^*^	0.057	0.067	-0.093
HDL-C (mmol/L)	0.002	-.145^**^	-.123^*^	0.060
LDL-C (mmol/L)	.189^**^	-0.012	0.056	0.054

Results given as Spearman correlation coefficient. SCZ, Schizophrenia; MDD, Major depressive disorder; HC, Healthy control; TC, Total cholesterol; TG, Triglycerides; HDL-C, High-density lipoprotein cholesterol; LDL-C, Low-density lipoprotein cholesterol; *p<0.05, **p <0.01. All associations were statistically significant. Bold values indicated statistical significance.

### 3.5 Factors influencing acute relapse in patients with schizophrenia and depression

To investigate the associations between inflammatory ratios (UHR, UAR, UCR, NHR, LHR, MHR, and PHR) and disease status, multivariate logistic regression analyses was conducted using variables identified in a preliminary univariate analysis. This approach enabled the identification of factors linked to the occurrence of SCZ and MDD while controlling for covariates, such as sex and age. In the binary logistic regression model, disease status served as the dependent variable, and age, sex, and the aforementioned inflammatory ratios were included as covariates. As detailed in **Table 4**, the binary logistic regression analysis for SCZ included UHR, UAR, NHR, and MHR, all of which was independently associated with SCZ. Multivariate analysis revealed that the UAR, NHR, and MHR were positively associated with SCZ, whereas UHR was negatively associated.

**Table 4:** Results of SCZ binary logistic regression analysis.

Variables	SCZ group		
	OR	95%CI	P
Age (year)	1.022	0.999-1.045	0.056
Sex (male/female)	0.697	0.420-1.156	0.162
UHR	0.987	0.983-0.991	**<0.001**
UAR	2.894	2.300-3.641	**<0.001**
NHR	1.354	1.051-1.745	**<0.05**
MHR*10	1.956	1.550-2.468	**<0.001**
Hosmer-Lemeshow Goodness of Fit Test		11.793(p=0.161)	

SCZ, Schizophrenia; UHR, Uric acid/HDL-C ratio; UAR, Uric acid/Albumin ratio; NHR, Neutrophil/HDL-C ratio; MHR, Monocyte/HDL-C ratio. Bold values indicated statistical significance.

For the analysis of MDD (**Table 5**), the binary logistic regression model included the UHR, UAR, UCR, MHR, and sex, all of which are independently associated with MDD. In the multivariate analysis, the UAR, the MHR, and sex were positively associated with MDD, whereas the UHR and UCR were negatively associated with MDD.

**Table 5:** Results of MDD binary logistic regression analysis.

Variables	MDD group		
	OR	95%CI	P
Age (year)	0.998	0.977-1.019	0.862
Sex (male/female)	1.986	1.067-3.699	**<0.05**
UHR	0.985	0.981-0.990	**<0.001**
UAR	4.041	3.030-5.388	**<0.001**
UCR	0.577	0.450-0.740	**<0.001**
LHR	1.046	0.670-1.632	0.878
MHR*10	1.916	1.535-2.390	**<0.001**
Hosmer-Lemeshow Goodness of Fit Test			12.674(p=0.124)

MDD, Major depressive disorder; UHR, Uric acid/HDL-C ratio; UAR, Uric acid/Albumin ratio; UCR, Uric acid/Creatinine ratio; MHR, Monocyte/HDL-C ratio. LHR, Lymphocyte/HDL-C ratio. Bold values indicated statistical significance.

*10 means multiplied by 10.

### 3.6 ROC curve analysis

**Figure 2:**
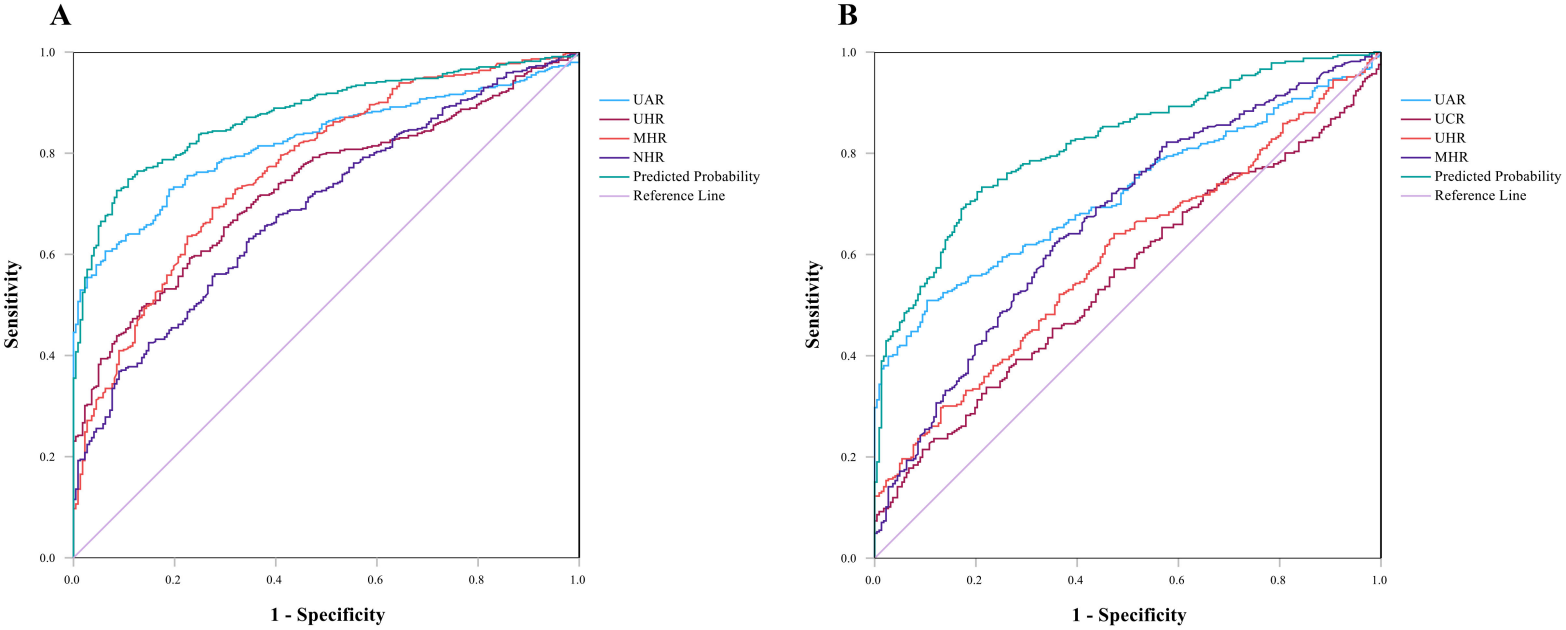
**(A)** ROC curves for the diagnostic ability of the parameters for SCZ (SCZ vs. HCs). UAR [AUC 0.822 (95% CI=0.791 to 0.853), p <0.001, critical value 8.515]; UHR [AUC 0.732 (95% CI=0.694 to 0.769), p <0.001, critical value 312.795]; MHR [AUC 0.771(95% CI=0.734 to 0.807), p <0.001, critical value 0.355]; NHR [AUC 0.692 (95% CI=0.651 to 0.732), p <0.001, critical value 2.720]. Indicator combination model (UAR + UHR+ NHR + MHR) [AUC 0.877 (95% CI=0.851 to 0.902), p<0.001, critical value 0.693]. **(B)** ROC curve for MDD, UAR [AUC 0.719 (95% CI=0.677 to 0.761), p <0.001, critical value 8.279]; UCR [AUC 0.554 (95% CI=0.586 to 0.689), p<0.05, critical value 6.709]; UHR [AUC 0.597 (95% CI=0.550 to 0.644), p<0.05, critical value 244.855]; MHR [AUC 0.669 (95% CI=0.624 to 0.715), p <0.05, critical value 0.327]. Indicator combination model (UAR +UCR+ UHR+ MHR) [AUC 0.818 (95% CI=0.784 to 0.853), p <0.001, critical value 0.597].

In the ROC curve for SCZ, parameters with an AUC greater than 0.6 included UHR [AUC 0.732 (0.694–0.769), p <0.001, cutoff 312.795, sensitivity 59.3%, specificity 77.00%], the UAR [AUC 0.822 (0.791–0.853), p <0.001, cutoff 8.515, sensitivity 60.60%, specificity 93.7%], the NHR [AUC 0.692 (0.651–0.732), p <0.001, cutoff 2.720, sensitivity 63.10%, specificity 65.30%], and the MHR [AUC 0.771 (0.734–0.807), p <0.001, cutoff 0.354, sensitivity 69.20%, specificity 72.50%]. The combined model of indicators improved diagnostic efficacy, with an AUC value of 0.877 [AUC 0.877 (0. 851–0.902), p <0.001, cutoff value 0.693, sensitivity 74.90%, specificity 89.20%]. The data are shown in **Figure 2A**.

ROC curve analysis of MDD revealed that indicators with AUCs greater than 0.6 were the UAR [AUC 0.719 (0.677–0.761), p <0.001, cutoff value 8.279, sensitivity 50.90%, specificity 89.60%] and the MHR [AUC 0.669 (0.624–0.715), p <0.05, cutoff value 0.327, sensitivity 63.20% and specificity 63.50%]. The AUCs less than 0.6 were the UCR [AUC 0.554 (0.586–0.689), p<0.05, critical value 6.709] and the UHR [AUC 0.597 (0.550–0.644), p<0.05, critical value 244.855]. The AUC value of the index joint model was 0.818 [AUC 0.818 (0.784–0.853), p <0.05, cutoff 0.597, sensitivity 73.30%, specificity 78.80%]. The data are shown in **Figure 2B**.

The effective predictive indicators for SCZ and MDD differ (AUC > 0.7); the former included UHR, UAR, and MHR, while the latter only included UAR. The AUC values for UAR, UHR, MHR, and the combined model in SCZ were all higher than those in MDD, which suggested that these indicators may have stronger predictive efficacy in SCZ.

## 3 Discussion

SCZ and MDD were believed to result from the interaction between genetic susceptibility and environmental factors, with inflammation and oxidative stress potentially contributing to their development. The recurrence rate of SCZ and MDD has drawn the attention of researchers (23, 24). This study demonstrated that the levels of potential novel biomarkers derived from UA and HDL-C vary significantly across different disease groups. Compared to the HC group, the SCZ group exhibited significantly higher levels of UHR, UAR, UCR, NHR, LHR, MHR, and PHR. In the MDD group, UAR, LHR, and MHR were significantly elevated compared to the HC group. Furthermore, the monocyte counts in both patient groups were higher than those in the HC group, while urea, TC, HDL-C, and albumin levels were all significantly lower. This indicated that patients with SCZ and MDD in this study may experience lipid metabolism disorders and varying degrees of inflammatory responses. ROC analysis results showed that UHR, UAR, NHR, and MHR could distinguish patients with SCZ from HC, with UHR and UAR exhibiting the highest specificity (>90%). Additionally, UAR and MHR could distinguish patients with MDD from healthy controls, with UAR demonstrating the highest specificity (89.6%). The predictive parameters for SCZ and MDD differ, and a combined model of these indicators could enhance diagnostic efficacy.

Corneal nerve morphology abnormalities in SCZ suggested that both neurodevelopmental and neurodegenerative processes were involved in the pathogenesis of SCZ (25). UA may exert a neuroprotective effect in this context by inhibiting oxidative stress and neuroinflammation (26). In this study, UA levels in patients with SCZ were significantly higher than those in the HC group (**Figure 1** and **Table 1**); however, this trend was not evident in patients with MDD. This finding has been corroborated by other studies (11), and UA levels increased following pharmacological treatment (27). Nevertheless, some studies contradict this finding (28, 29). Given the complex role of UA and the heterogeneity among studies, further cohort studies are needed to explore the relationship between UA levels and the onset and progression of SCZ and MDD. Creatinine and albumin were endogenous antioxidants with free radical scavenging functions (27, 30). Consequently, combined indicators of UA, creatinine, and albumin may provide a more accurate reflection of inflammation and oxidative stress levels than individual parameters alone (16). Studies have shown that serum albumin levels are depleted during both the acute and remission phases of SCZ (27), which was consistent with our research. ROC curve analysis showed that the UAR and UCR were independent predictive factors for distinguishing patients with SCZ or MDD from HCs. These findings were consistent with results from studies on manic episodes in patients with bipolar disorder (BD) and associated with psychiatric symptoms (16). Currently, few studies have explored the UAR, UCR, and ULR in patients with mental disorders, and whether they are protective or risk factors for mental disorders remains to be further investigated.

HDL-C was associated with clinical symptoms of SCZ and MDD, suggesting its potential significance as a biomarker (31–33). In patients with TRS, HDL-C levels were significantly lower than those in the HC group (11), and baseline levels may serve as a potential indicator for predicting the improvement of depressive symptoms in female MDD patients (34). However, the patterns of change in other lipid indicators such as TC, LDL-C, and TG differ between SCZ and MDD, and the research findings are not entirely consistent (35, 36). This heterogeneity may arise from various confounding factors, including medications (37), genetics (38), environment, and lifestyle (39), and differences between studies and disease subtypes (40). Among these factors, the impact of medications on lipid changes is limited and specific to certain lipids, suggesting that alterations in the lipid profile may be inherent characteristics of these mental disorders rather than merely a consequence of drug side effects (40). Therefore, the precise relationship and causal mechanisms between lipid abnormalities and mental disorders necessitate further investigation.

In recent years, the ratio related to HDL-C has emerged as a novel indicator, demonstrating significant value in the risk assessment, diagnosis, and prediction of various diseases. Currently, there is no research on UHR in SCZ. Our study found that UHR levels in the SCZ and MDD groups were higher than those in the HC group (**Table 1**, p<0.05). A negative correlation was observed between UHR and both SCZ and MDD. However, existing studies on the relationship between UHR and MDD have shown controversy, with some indicating a positive correlation (29, 41) while others suggesting a negative correlation (42). These discrepancies may be influenced by confounding factors such as gender (21, 43) and ethnicity. Therefore, further validation of the relationship between UHR and mental disorders, as well as its clinical significance, is necessary through multicenter or stratified studies. The NHR, LHR, MHR, and PHR serve as comprehensive indicators of systemic inflammation, integrating HDL-C and parameters from complete blood counts, which include neutrophil, lymphocyte, monocyte, and platelet counts. Within the intricate network of interactions between immune cells and HDL-C, the combination of comprehensive indicators such as LHR, MHR, and PHR provided a more systematic assessment of inflammation than individual parameters (44). In this study, we identified NHR and MHR as predictive factors for SCZ, while MHR was effective in distinguishing between MDD patients and HCs. These findings are consistent with previous research (20, 44), reinforcing the application value of NHR, LHR, MHR, and PHR as novel inflammatory markers in patients with SCZ and MDD.

We found that there were different levels of association between lipid indicators and blood immune cells (**Table 3**). This result was consistent with earlier research as well as our current investigation (45). The innate immune system’s main defenses against infection include neutrophils, monocytes, lymphocytes, and platelets. Because it suppresses the activation of immune cells like neutrophils and macrophages and controls anti-inflammatory pathways, HDL-C was crucial for preserving immunological homeostasis (14). Additionally, alterations in markers associated with platelets suggested that platelet activation might impact the pathophysiological mechanisms of MDD and SCZ by triggering inflammatory reactions, releasing pro-inflammatory factors, and altering neurotransmitters, thus intensifying the symptoms and course of these disorders (11, 34). Interestingly, obesity and hyperlipidemia were more prevalent among patients with MDD (46), which was associated with cognitive decline, reduced gray matter volume, and impaired white matter integrity (47). In patients with SCZ, BMI was negatively correlated with cognitive function (48). BMI, a commonly used indicator for assessing obesity, is closely related to lipid metabolism abnormalities when increased. Therefore, BMI is crucial in the exploration of new biomarkers, and it may be necessary to conduct further stratified studies. Additionally, the impact of medications should not be overlooked (49).

This study presents both novel contributions and acknowledged limitations. In contrast to previous research that has primarily focused on a single disease or isolated biomarkers (17–19, 50), we utilized retrospective clinical data to analyze eight potential biomarkers derived from UA, HDL-C, and blood cell counts. These easily accessible laboratory indicators reflect evidence of oxidative balance metabolic disorders, and inflammatory responses during the progression of SCZ and MDD. We propose and develop a composite model to assess the clinical predictive performance of these potential biomarkers. Notably, these potential biomarkers can be used in conjunction with emerging biomarkers, such as microRNAs and corneal confocal microscopy (25, 51) with the expectation that further research will validate these findings. However, this study has limitations. First, all of the study participants were acute relapse inpatients, and the potential impact of medication on these markers was not considered. Second, factors such as diet, BMI, smoking, alcohol consumption and lifestyle were not considered, even though these factors may influence changes in these markers. Third, validated scales were not used to measure the severity of psychiatric symptoms, and these were not included in the assessment. Fourth, this was a single-center retrospective study that lacks external data validation. Therefore, no clear causal relationship can be inferred. Given the significant gender differences and multiple limitations of the data in this study, we aim to provide some insights for potential biomarker research, whose predictive value for clinical outcomes (such as recurrence or treatment response) has not yet been verified. More clinical studies are needed to further validate our findings.

## 4 Conclusions

This study conducted a retrospective comparative analysis of eight potential biomarkers, revealing significant abnormalities in inflammatory markers among patients with SCZ and MDD. Monocytes were notably elevated, while TC, HDL-C, and albumin were significantly reduced. Significant differences were observed in UHR, UAR, LHR, and MHR among the SCZ, MDD, and control groups. UHR, UAR, NHR, and MHR could serve as predictive factors for distinguishing SCZ patients from HCs. Additionally, UHR, UAR, UCR, and MHR may function as predictive factors for differentiating severe MDD patients from HCs. The combination of these indicators significantly enhances predictive efficacy. In the future, these biomarkers, associated with inflammation and oxidative stress pathways, may serve as valuable adjuncts in the diagnosis and differential diagnosis of SCZ and MDD. This study provides an important reference for future longitudinal research.

## Data Availability

The original contributions presented in the study are included in the article/supplementary material. Further inquiries can be directed to the corresponding author.
